# Polycomb Target Genes Are Silenced in Multiple Myeloma

**DOI:** 10.1371/journal.pone.0011483

**Published:** 2010-07-09

**Authors:** Antonia Kalushkova, Mårten Fryknäs, Miguel Lemaire, Charlotte Fristedt, Prasoon Agarwal, Maria Eriksson, Sarah Deleu, Peter Atadja, Anders Österborg, Kenneth Nilsson, Karin Vanderkerken, Fredrik Öberg, Helena Jernberg-Wiklund

**Affiliations:** 1 Rudbeck Laboratory, Department of Genetics and Pathology, Uppsala University, Uppsala Sweden; 2 Department Hematology and Immunology, Vrije Universiteit Brussel (VUB), Brussels, Belgium; 3 Novartis Institute for Biomedical Research, Cambridge, Massachusetts, United States of America; 4 Department of Hematology, Karolinska University Hospital Solna, Stockholm, Sweden; Ludwig-Maximilians-Universität München, Germany

## Abstract

Multiple myeloma (MM) is a genetically heterogeneous disease, which to date remains fatal. Finding a common mechanism for initiation and progression of MM continues to be challenging. By means of integrative genomics, we identified an underexpressed gene signature in MM patient cells compared to normal counterpart plasma cells. This profile was enriched for previously defined H3K27-tri-methylated genes, targets of the Polycomb group (PcG) proteins in human embryonic fibroblasts. Additionally, the silenced gene signature was more pronounced in ISS stage III MM compared to stage I and II. Using chromatin immunoprecipitation (ChIP) assay on purified CD138+ cells from four MM patients and on two MM cell lines, we found enrichment of H3K27me3 at genes selected from the profile. As the data implied that the Polycomb-targeted gene profile would be highly relevant for pharmacological treatment of MM, we used two compounds to chemically revert the H3K27-tri-methylation mediated gene silencing. The S-adenosylhomocysteine hydrolase inhibitor 3-Deazaneplanocin (DZNep) and the histone deacetylase inhibitor LBH589 (Panobinostat), reactivated the expression of genes repressed by H3K27me3, depleted cells from the PRC2 component EZH2 and induced apoptosis in human MM cell lines. In the immunocompetent 5T33MM *in vivo* model for MM, treatment with LBH589 resulted in gene upregulation, reduced tumor load and increased overall survival. Taken together, our results reveal a common gene signature in MM, mediated by gene silencing via the Polycomb repressor complex. The importance of the underexpressed gene profile in MM tumor initiation and progression should be subjected to further studies.

## Introduction

Multiple myeloma (MM) remains a fatal hematopoietic malignancy. MM is characterized by the clonal expansion of tumor cells with plasma cell features in the bone marrow and is considered a genetically heterogeneous disease [1]. Although the predominant translocations in MM do not fully explain the pathogenesis of the malignant plasma cell, the identification of genetic entities has facilitated the development of targeted therapy, such as FGFR3-kinase inhibitors in t(4;14) MM [2]. However, such treatment does not apply to all MM patients. New therapeutic strategies targeting common pathogenetic events in MM are therefore imperative.

An emerging strategy to fight cancer complexity is to establish gene expression profiles and connect them to specific signaling pathways contributing to the disease [3]. In MM, gene expression profiles have, in some cases, been linked to an underlying genetic event such as a translocation to the immunoglobulin heavy-chain locus or hyperdiploidy [4], [5]. In further attempts to dissect the disease phenotype, gene expression profiles have been used to refine the underlying mechanisms in molecular subsets, to discover predictors of drug response and identify novel drug targets [6], [7], [8], [9]. However, it still remains unclear how MM, although phenotypically representing mature plasmablasts/plasma cells, preserves the capacity of self-renewal and whether this capacity may be identified as a common gene expression signature.

Recently, components of the Polycomb group (PcG) proteins have gained a wide interest as prominent players in carcinogenesis [10], [11]. The PcG proteins function in large multimeric complexes, of which the Polycomb repressive complexes PRC1 and PRC2 are most well characterized. The PRC2 core complex consists of EED, SUZ12, RBAP48 and the catalytic subunit EZH2 [12]. PcG mediated gene repression requires a complex series of events, initiated by the recruitment of PRC2 to target genes resulting in the tri-methylation of histone H3K27, which then preserves silencing of the transcriptional program through consecutive cell divisions [13]. PcG mediated silencing is also suggested to predispose target genes to DNA-methylation in various cancers [14], [15], [16] consistent with the observation that the PRC2 complex interacts with DNMT1 and DNMT3A and B [17]. However, PcG mediated repression may also constitute an independent mechanism of silencing for some cancer genes, in the absence of DNA-methylation [18].

In the present study integrative genomics was used to define the nature of the underexpressed gene expression profile in MM when compared to normal plasma cells. The identified gene signature significantly correlated to defined Polycomb target genes [19] and was more pronounced in advanced stages of the disease. Enrichment of H3K27me3 at genes found in the profile was confirmed by chromatin immunoprecipitation (ChIP) assay in four newly diagnosed MM patients and two MM cell lines. The S-adenosylhomocysteine hydrolase inhibitor 3-Deazaneplanocin (DZNep) and the histone deacetylase inhibitor LBH589 (Panobinostat) reactivated the genes repressed by H3K27me3, depleted cells of the PRC2 component EZH2, reduced proliferation and increased apoptosis in human MM cell lines. Using the immunocompetent 5T33MM *in vivo* model we show gene upregulation, reduction of tumor load and increased overall survival by LBH589. Collectively, our results point to a common gene expression signature in MM mediated by gene silencing via the PRC2 complex. Unraveling the underlying molecular mechanisms and the biological significance of reactivating the genes found in this signature may have new therapeutic implications.

## Results

### H3K27-tri-methylation is a common denominator for the underexpressed genes in multiple myeloma (MM)

**Figure 1:**
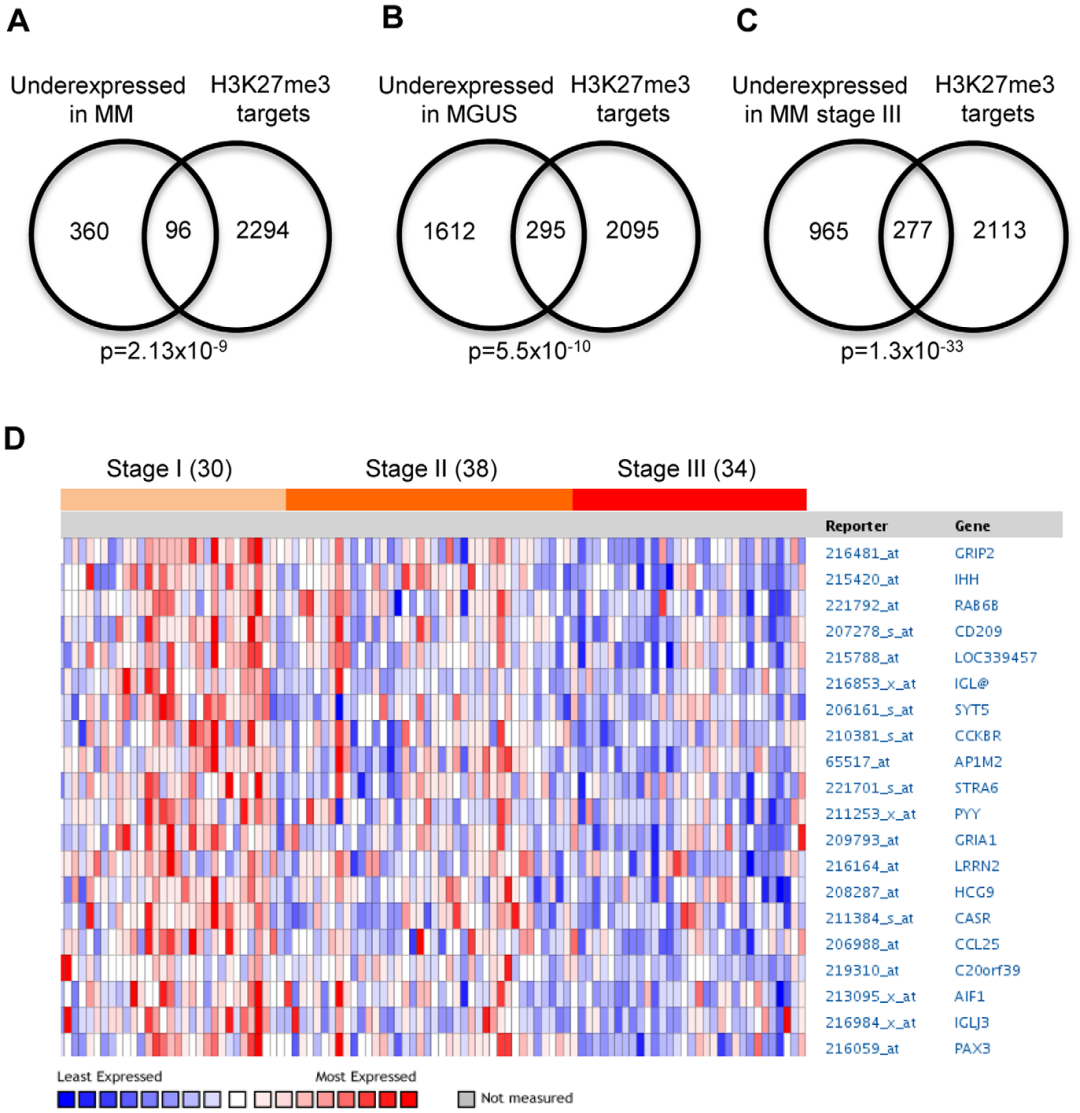
H3K27me3 targets are underexpressed in multiple myeloma (MM) and monoclonal gammopathy of undetermined significance (MGUS). The H3K27me3 target genes (n = 2390) were defined by Bracken et al. [19] in embryonic fibroblasts and found to be statistically associated to underexpressed genes in MM and MGUS using Oncomine. (A): The top 10% underexpressed genes [7] (n = 456) in MM patients (n = 74) compared to plasma cells (n = 37) and tonsillar tissue (n = 37) showed significant (p = 2.13×10^−9^) overlap with the H3K27me3 target genes. (B): The top 10% underexpressed genes [20] (n = 1907) in MGUS patients (n = 44) compared to normal bone marrow (n = 22) showed significant (p = 5.5×10^−10^) overlap with the H3K27me3 target genes. (C): The top 10% underexpressed genes [5] (n = 1242) in MM stage III (n = 34) compared to MM stage I and stage II (n = 68) showed significant (p = 1.3×10^−33^) overlap with the H3K27me3 target genes. (D): H3K27me3 target genes expression during MM progression [5].

Publicly available gene expression data sets [5], [7], [20] were analyzed in an integrative genomics approach to identify unifying denominators among genes underexpressed in multiple myeloma (MM). Using the data-mining platform Oncomine [21] a strong connection between genes underexpressed in patients with MM (compared to normal bone marrow) and genes previously described as H3K27me3 targets in human embryonic fibroblasts [19] was discovered (Figure 1A, for a complete gene list see Table S1). The H3K27me3 target genes were also overrepresented among genes underexpressed in MGUS patients (Figure 1B) and strongly associated with decreased expression in ISS stage III MM (Figure 1C), compared to stage I and II (Figure 1D). Consistent with this, overexpression of the PRC2 components EZH2, SUZ12 and EED was significantly correlated with the establishment and progression of MM (Figure S1A–C).

### Polycomb target genes are H3K27 tri-methylated in patient MM cells and MM cell lines

**Figure 2:**
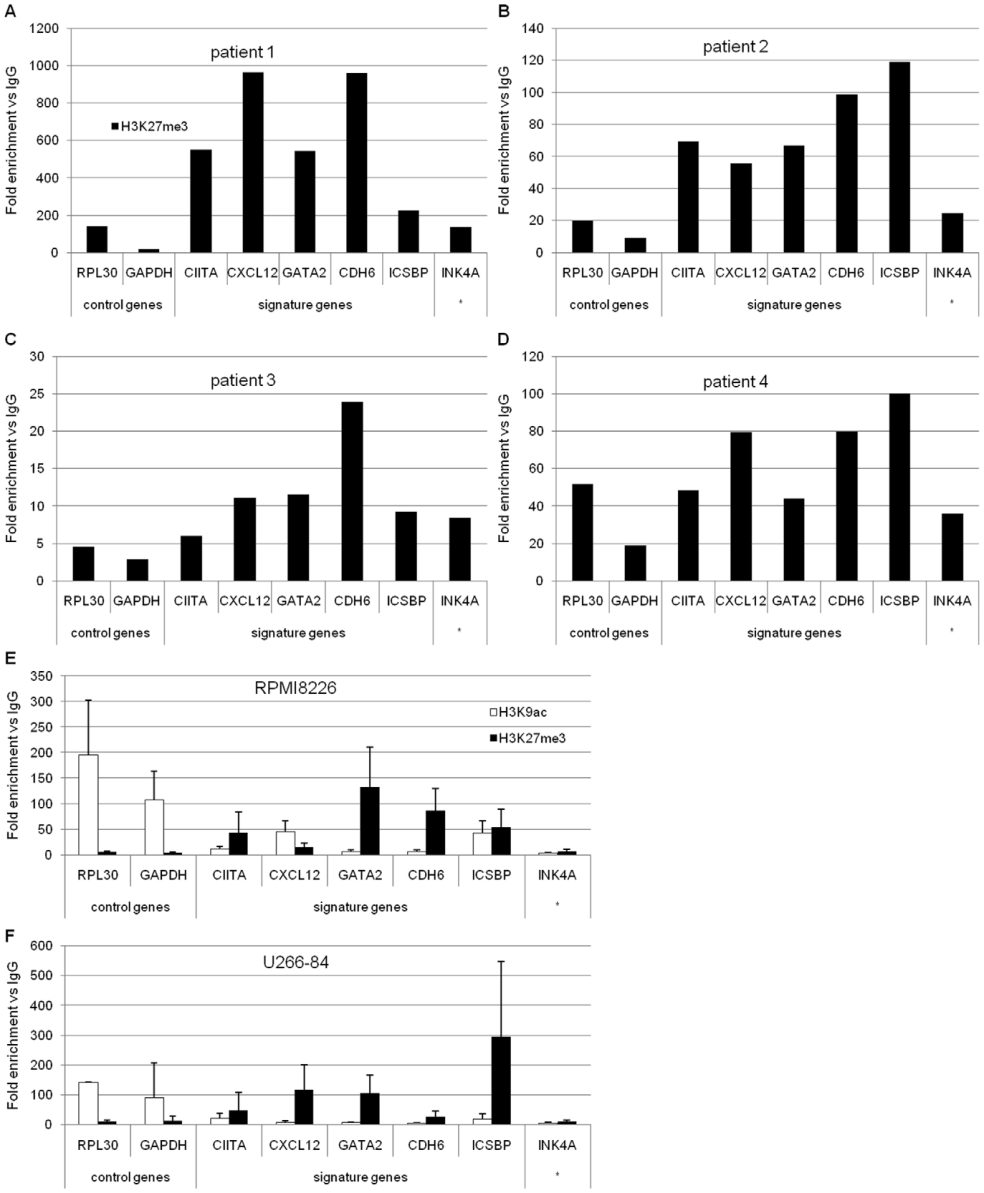
Polycomb target genes are enriched for H3K27-tri-methylation in MM patient cells and MM cell lines. Chromatin immunoprecipitation (ChIP) assay using (A)–(D): antibody against H3K27-tri-methylation in purified CD138+ cells from MM patients; (E) and (F): antibodies against H3K27-tri-methylation and H3K9-acetylation in RPMI 8226 and U-266-1984 cells. The RPL30 and GAPDH genes were used as a negative control for H3K27-tri-methylation and a positive control for H3K9-acetylation. The CIITA, CXCL12, GATA2, CDH6 and ICSBP genes were selected from the integrative genomics profile. * The INK4A gene was selected from the literature. Immunoprecipitated DNA was analyzed using real-time qPCR. Specific signal was calculated as fold change between signal and background (IgG) noise normalized to percent input. (E) and (F): Error bars represent SD from three independent biological experiments.

Having established that the underexpressed gene profile in MM significantly overlaps with defined Polycomb target genes, the PRC2-mediated tri-methylation of histone H3 lysine 27 was analyzed by chromatin immunoprecipitation (ChIP) in four newly diagnosed MM patients (Figure 2A–D). Considering the limited availability of patient material, enrichment for the negative H3K27-tri-methylation and positive H3K9-acetylation marks was analyzed in the RPMI 8226 (Figure 2E) and U-266-1984 (Figure 2F) MM cell lines. The chromatin modifications were determined at five PRC2 targets commonly underexpressed in MM (CIITA, CXCL12, GATA2, CDH6 and ICSBP/IRF8; Figure S2A–C). The selected genes were then additionally confirmed to be underexpressed in MM compared to normal counterpart plasma cells in an independent data set [22] (GSE6477, Figure S3). The INK4A/p16 gene was also included, as a previously reported target of the Polycomb group (PcG) proteins [23]. The actively transcribed genes RPL30 and GAPDH were used as control genes for the lack of H3K27-tri-methylation and presence of acetylation on histone H3 lysine 9.

Consistent with the role for Polycomb-mediated silencing, the analyzed genes were found enriched for H3K27-tri-methylation in the four MM patient samples (Figure 2A–D) and both MM cell lines RPMI 8226 (Figure 2E) and U-266-1984 (Figure 2F). These genes also lacked the positive chromatin acetylation mark at H3K9 in both MM cell lines. An exception to this was the ICSBP/IRF8 gene, which was clearly enriched for H3K27-tri-methylation in two of the four patients and the U-266-1984 cell line, whereas in the RPMI 8226 cells carried both the positive and negative mark. The CIITA gene lacked clear enrichment in one of the patients and the CXCL12 gene carried the positive H3K9-acetylation mark in the RPMI 8226 cells and the negative H3K27-tri-methylation mark in the U-266-1984 cells.

### The S-adenosylhomocysteine hydrolase inhibitor 3-Deazaneplanocin (DZNep) and the histone deacetylase inhibitor LBH589 reactivate Polycomb target genes in MM cell lines

We next investigated the possibility of reactivating the genes enriched for H3K27-tri-methylation. The expression of the genes selected from the integrative genomics profile was analyzed after treatment with the chemical inhibitors, DZNep and LBH589, in the RPMI 8226 and U-266-1984 cell lines. RPMI 8226 and U-266-1984 were treated with 10 µM DZNep and 20 nM LBH589 and the expression of CIITA, CXCL12, GATA2, CDH6, ICSBP/IRF8 and INK4A was analyzed using Q-RT-PCR after 6, 24, 48 and 72 hours.

**Figure 3:**
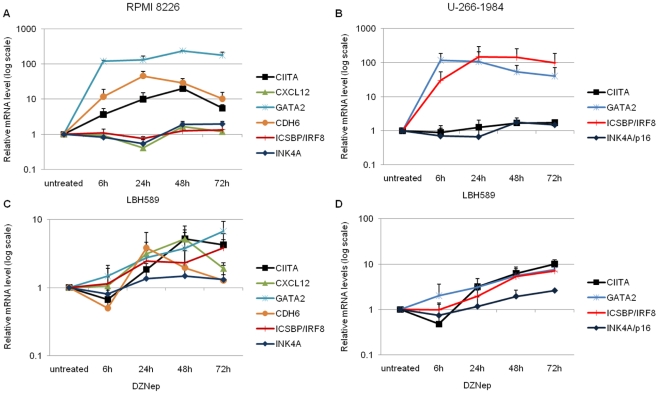
The expression of H3K27-tri-methylated genes is reactivated by DZNep and LBH589 in MM cell lines. The RPMI 8226 cells (A) and (C), and the U-266-1984 cells (B) and (D) were treated with DZNep (10 µM) or LBH589 (20 nM) for 6, 24, 48 and 72 hours. Fold difference in expression was calculated relative to the untreated cells. GAPDH was used as a reference gene. Error bars represent SD from three independent biological experiments.

LBH589 (Figure 3A and 3B) was more potent and led to a higher increase in mRNA levels at earlier time points in both cell lines, whereas, DZNep (Figure 3C and 3D) increased mRNA levels to a lesser extent and at later time points. Changes in gene expression varied between the cell lines and had slightly different kinetic profiles. CIITA mRNA expression in RPMI 8226 increased after DZNep and LBH589 treatment. In U-266-1984, CIITA expression gradually increased only by DZNep. In RPMI 8226, CXCL12 gene expression was upregulated only after DZNep treatment. GATA2 responded strongly to both drugs in both cell lines. The CDH6 mRNA level was increased by both DZNep and LBH589 in RPMI 8226. ICSBP/IRF8 expression increased gradually by DZNep in both cell lines; LBH589 strongly increased ICSBP/IRF8 mRNA levels only in U-266-1984. INK4A/p16 responded weakly to both drugs in both cell lines.

CXCL12 and CDH6 mRNA levels were detected at very late amplification cycles in U-266-1984 and were not amenable to analysis.

### Both DZNep and LBH589 deplete EZH2 protein levels in MM cell lines

EZH2 is the catalytically active subunit of PRC2 responsible for the establishment of tri-methylation at histone H3 lysine 27 that is associated with gene silencing [24]. The two chemical inhibitors, the S-adenosylhomocysteine hydrolase inhibitor 3-Deazaneplanocin (DZNep) and the histone deacetylase inhibitor LBH589, are reported to deplete EZH2 protein levels and reduce survival in cancer cells [25], [26].

**Figure 4:**
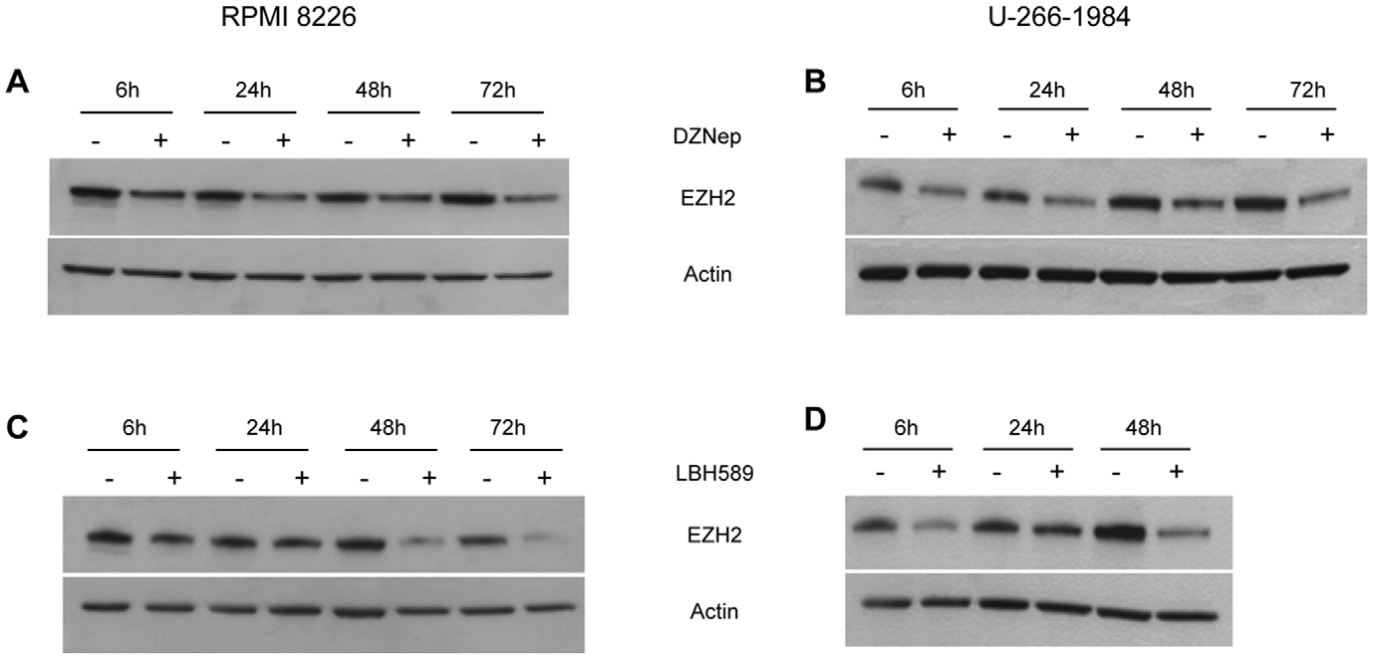
DZNep and LBH589 deplete EZH2 protein level in MM cell lines. (A) and (B): The RPMI 8226 cells (A) and the U-266-1984 cells (B) were treated with DZNep (10 µM) for 6, 24, 48 and 72 hours. (C) and (D): The RPMI 8226 cells (C) and the U-266-1984 cells (D) were treated with LBH589 (20 nM) for 6, 24, 48 and 72 (RPMI 8226) or 6, 24 and 48 hours (U-266-1984). Western blot was performed using a specific antibody against EZH2. Actin was used to control for equal loading.

The MM cell lines, RPMI 8226 and U-266-1984 were treated with DZNep at 0.5, 2.5 and 10 µM or LBH589 at 4, 10, 20 nM (RPMI 8226) and 4, 20 and 100 nM (U-266-1984). Depletion of EZH2 protein levels measured by Western blotting analysis was observed in a dose-dependent manner for both drugs in both cell lines (Figure S4A–D). Treatment with 10 µM DZNep for 6, 24, 48 and 72 hours led to a gradual depletion of EZH2 protein in both the RPMI 8226 and the U-266-1984 cells (Figure 4A and 4B). Additionally, the EZH2 protein level was reduced after treatment with 20 nM LBH589 with the strongest reduction observed at the latest time point of treatment, 48 hours for U-266-1984, and 72 hours for RPMI 8226 (Figure 4C and 4D).

### Both DZNep and LBH589 independently inhibit growth of MM cell lines

**Figure 5:**
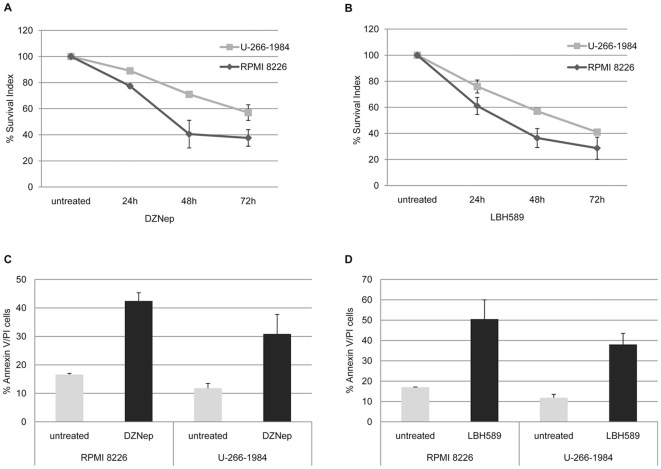
DZNep and LBH589 reduce growth and induce apoptosis in MM cell lines. (A) and (B): U-266-1984 and RPMI 8226 cells were treated for 6, 24, 48 and 72 hours with DZNep (10 µM) or LBH589 (20 nM) followed by a resazurin assay. At least 2 independent experiments were performed in triplicates, data is presented as mean percentage of control ±SD. (C) and (D): U-266-1984 and RPMI 8226 were treated with DZNep (10 µM) and LBH589 (20 nM) for 72 hours followed by AV/PI staining and flow cytometry analysis. At least 2 independent experiments per cell line were performed; data is presented as mean percentage apoptotic cells ±SD.

To elucidate the effects of Polycomb inhibitors on the growth and survival of MM cells, RPMI 8226 and U-266-1984 were treated with DZNep or LBH589 for 6, 24, 48 and 72 hours and cell growth was analyzed by the resazurin assay. Both treatments decreased the relative number of viable MM cells in a dose-dependent manner (Figure S5A and S5B). On the contrary, treatment with the same concentrations of both drugs of normal peripheral blood mononuclear cells (PBMC) and normal human fibroblast SK 1064 cells showed no decrease in cell viability (Figure S6A and S6B). Treatment with 10 µM DZNep exhibited over 60% growth inhibition in RPMI 8226 and over 40% in U-266-1984 at 72 hours of treatment, compared to the untreated cells (Figure 5A). Treatment with 20 nM LBH589 was more potent in reducing the relative amount of viable cells and led to a 70% and 60% growth inhibition at 72 hours in RPMI 8226 and U-266-1984, respectively (Figure 5B).

To further characterize the effect of DZNep and LBH589 in reducing the number of viable cells, induction of apoptosis was quantified by AnnexinV/PI staining using FACS analysis. At 72 hours DZNep treatment increased the fraction of late apoptotic/necrotic cells in RPMI 8226 and U-266-1984 more than two fold (Figure 5C). Treatment with LBH589 was more potent in inducing apoptosis and led to approximately 3 fold increase of AnnexinV/PI positive apoptotic cells in RPMI 8226 and greater than 3 fold increase in U-266-1984 cells at 72 hours (Figure 5D).

### LBH589 treatment upregulates Polycomb target gene expression, decreases tumor load and promotes survival in the murine 5T33MM model

**Figure 6:**
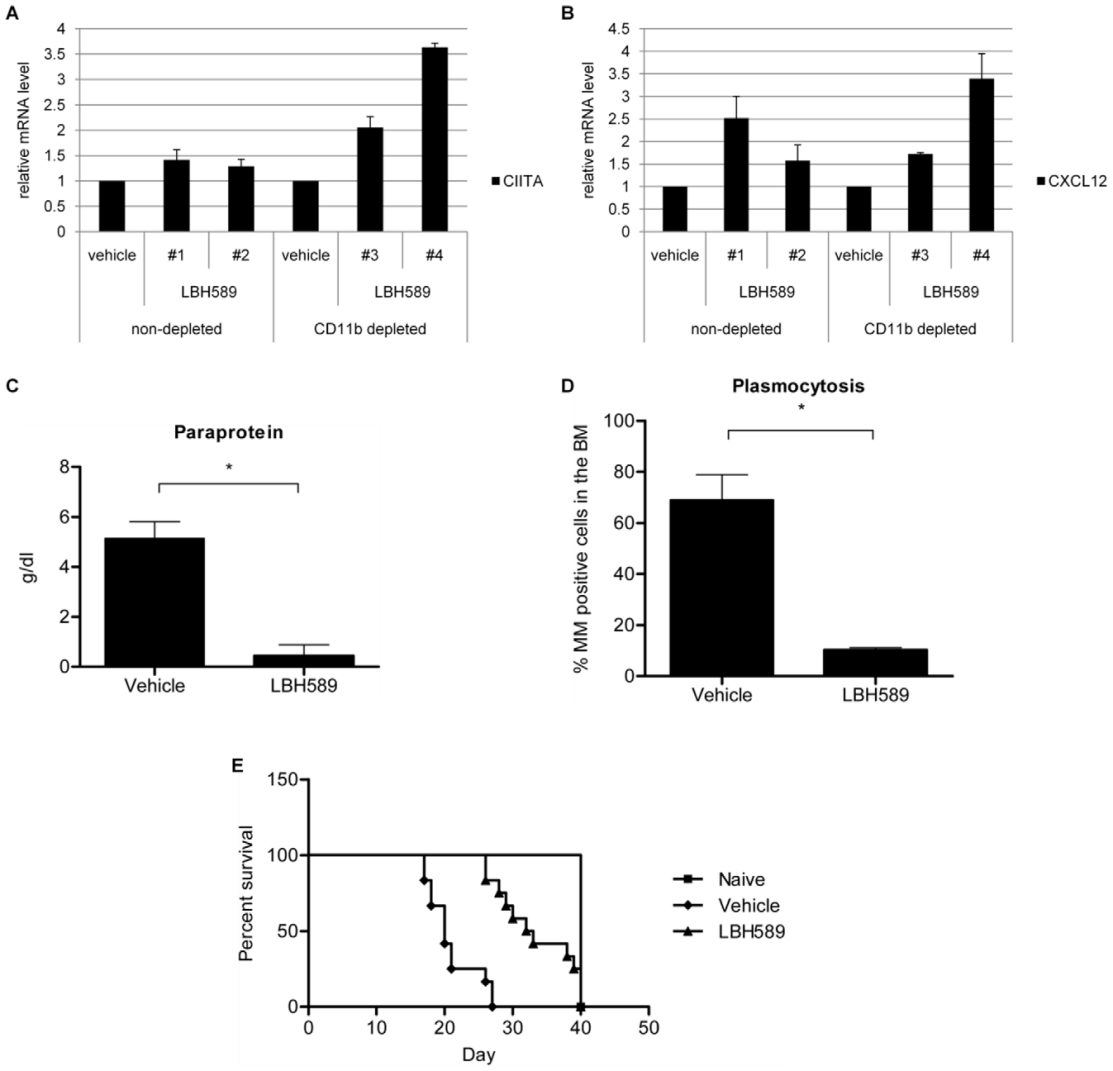
LBH589 upregulates gene expression, reduces tumor load and increases survival in the 5T33MM mice model. (A) and (B): At day 0 C57BL/KalwRij mice were injected with 0.5×10^6^ 5T33MM cells. After 14 days mice were either assigned to a treatment group receiving 10 mg/kg LBH589 (n = 4; daily i.p. injection) or to a vehicle group receiving 0.9% NaCl solution (n = 4; daily i.p. injection). After 5 days of treatment mice were sacrificed, BM harvested and a negative selection for CD11b positive cells performed. CIITA and CXCL12 fold difference in gene expression was calculated relative to the vehicle by Q-RT-PCR. GAPDH was used as a reference gene. The error bars represent SEM from two independent Q-RT-PCR runs. Each group contained one mouse, excluding the vehicle non-depleted, which contained two mice. (C) and (D): Mice were treated as described above, with the difference that mice were either assigned to a treatment group receiving 10 mg/kg LBH589 (n = 10; daily i.p. injection), to a vehicle group receiving 0.9% NaCl solution (n = 10; daily i.p injection) or to an untreated, disease-free group (n = 10) as a control group at day 0. The animals were sacrificed when the vehicle group showed signs of morbidity. Tumor load as determined by serum electrophoresis (C) and tumor load as determined by May Grünwald-Giemsa staining of BM cytospin samples (D) Mean values ± SD for groups of 10 mice are shown (*p<0.0001). (E) Survival was studied using the Kaplan-Meier analysis method. Mice were treated as described above with the exception that treatment continued until each animal showed signs of morbidity. Each group contained 12 mice (p<0.0003).

Similar to human MM cell lines, treatment with LBH589 induces decreased DNA synthesis, cell cycle arrest and apoptosis in 5T33MM cells *in vitro* (data not shown). Here, the murine syngeneic immunocompetent 5T33MM model [27], [28] was used to evaluate the effect of LBH589 treatment *in vivo*. The C57BL/KalwRij mice were injected with the myeloma 5T33MM cells and after 14 days subjected to treatment with LBH589 (10 mg/kg) as described in materials and methods. After 5 days of treatment mice were sacrificed and their BM harvested. To confirm the reactivation of CIITA and CXCL12 gene expression in the *in vivo* 5T33MM model, RNA was extracted from the total BM fraction and from CD11b-depleted fraction of BM cells. We found that CIITA was upregulated after LBH589 treatment only in the CD11b-depleted bone marrow cells (Figure 6A), whereas CXCL12 was upregulated both in the crude BM cell fraction and in the CD11b-depleted cells (Figure 6B). Secondly, the effect of LBH589 as single drug treatment (continuous treatment) was determined on the *in vivo* tumor burden. The C57BL/KalwRij mice were injected with 5T33MM cells and the effect of LBH589 treatment compared to a vehicle treatment was analyzed. A significant decrease (p<0.0001) in the tumor load after LBH589 treatment was determined by the amount of detected paraprotein (M spike, Figure 6C) and the percentage of MM positive cells in isolated bone marrow cells (Figure 6D). Finally, LBH589 treatment significantly increased (p<0.0003) the survival of the 5T33MM mice, as displayed by the Kaplan-Meier survival curve (Figure 6E).

## Discussion

The genetic heterogeneity of multiple myeloma (MM) [1] has hindered the identification of the mechanisms underlying its pathogenesis and hence, MM remains a fatal disease. Both the translocations to the immunoglobulin heavy chain (IgH) locus and the chromosomal trisomies observed in MM fail to explain a common origin of the disease and to provide a universal MM drug target. Gene expression profiles have provided clues of the importance of previously unrecognized signaling pathways in MM such as the NF-κB pathway [6], [8], but to date have mainly focused on subdividing MM into genetic entities for prognostic or therapeutic purposes [9], [20]. Thus, the question remains if a common denominator for myeloma development and tumor maintenance exists.

By means of integrative genomics we identified a set of underexpressed genes in MGUS and MM patient cells relative to normal bone marrow plasma cells and total bone marrow cells, respectively. These genes have also been previously described as targets of the Polycomb group (PcG) proteins in human embryonic fibroblasts [19]. Importantly, the suppression of the genes found in the profile was more pronounced in the advanced stages (ISS stage III compared to stage II and I) of MM progression.

The presence of tri-methylation at histone H3 lysine 27 using chromatin immunoprecipitation (ChIP) was determined for several genes found in the profile in four MM patient samples, and two MM cell lines RPMI 8226 and U-266-1984. Among the four patients the genes selected from the integrative genomics approach were consistently enriched for H3K27me3. In addition, simultaneous enrichment for the negative H3K27me3 and lack of the H3K9-acetylation mark associated with an active transcription, suggested active Polycomb-mediated silencing also in the two MM cell lines. Notably, among the genes found in the expression profile, and associated with H3K27me3, was the major histocompatibility (MHC) class II transactivator (CIITA) gene. CIITA is the master regulator of MHC class II expression. Underexpression of CIITA and thus of MHC II genes in MM has been described as a mechanism to escape the immune response [29]. Independently, Polycomb silencing of CIITA is described in uveal melanoma [30]. Another gene present in the profile was ICSBP/IRF8, which frequently displays low levels of expression in patient plasma cells and MM cell lines. This silencing mechanism is attributed to DNA methylation of the ICSBP/IRF8 gene in 8 out of 10 MM cell lines, including U-266-1984, whereas the frequency is lower in CD138+ selected primary MM cells with only 1 out of 9 being methylated [31]. In the present study ICSBP/IRF8 was associated with H3K27me3-mediated silencing in 3 out of 4 patients and the U-266-1984 cell line. Interestingly, DNA methyltransferases are known to be associated with the PRC2 complex [17], and thus DNA methylation following Polycomb-mediated gene silencing is suggested to contribute to permanent transcriptional repression in cancer [14], [15], [16], [18]. Hence, PcG mediated repression may be sufficient, and in some cases precede DNA methylation and explain the observed ICSBP/IRF8 silencing in MM patients where DNA methylation is not detected. Our results are also consistent with a previous study suggesting EZH2 as an oncogene in MM, where it is often overexpressed and contributes to cell survival [32]. An alternative mechanism for the deregulation of Polycomb control of gene expression in MM was recently suggested by the finding of prevalent mutations in the H3K27-demethylase UTX [33].

The identification of the Polycomb repression gene profile based on differentially expressed genes in MM patient cells, strongly suggests that this profile is a common feature among the tumor cells, rather than representing a specific subpopulation. Thus, an emerging question is if targeting the common denominator for the observed gene silencing can be used in the treatment of MM. Epigenetic gene silencing is an attractive drug target due to the fact it can be reverted. However, Polycomb targets are not uniformly reactivated solely by depleting cells of PcG proteins using siRNA interference [18], [19], [26], [34]. In this study, two chemical inhibitors, the global histone methylation inhibitor 3-Deazaneplanocin (DZNep) [26], [35] and the histone deacetylase inhibitor LBH589, were successfully used to reactivate selected genes from the profile, which carried the H3K27me3 mark, including CIITA and INK4A/p16. Moreover, gene upregulation by LBH589 treatment *in vivo* was also observed in the 5T33MM model of MM. In addition to their role on global chromatin modifications and their ability to reactivate Polycomb-silenced genes, DZNep and LBH589 have also been reported to deplete EZH2 protein from AML and breast cancer cells [25], [26]. Similarly, both DZNep and LBH589 depleted EZH2 protein also from MM cells. According to recent data LBH589 treatment of cancer cells leads to ubiquitin-dependent degradation of DNMT1 [36]. However, further studies are needed to clarify whether a similar mechanism may contribute to the protein downregulation of EZH2 in MM cells.

In addition to the reactivation of the Polycomb-target genes both DZNep and LBH589 reduced viability and induced apoptosis in two MM cell lines. As LBH589 has an advantageous efficiency at nanomolar concentrations and is currently in phase I and II clinical trials for several hematologic malignancies including MM [37]; its effects on MM growth were evaluated also *in vivo* using the murine 5T33MM model. Treatment with LBH589 significantly decreased tumor load and levels of paraprotein. Additionally, LBH589 prolonged survival in the murine myeloma 5T33MM model. Thus, our data strongly supports the use of LBH589 in the clinics and provides and alternative mechanism for its antitumor action. Although limited effects of LBH589 single administration have been reported, LBH589 is currently in phase I clinical trials in combinatorial regimens for relapsed MM [38] due to the fact that it is able to overcome drug resistance in MM cell lines and cells from patients with refractory disease [39]. Recently, a combination of both LBH589 and DZNep was reported to be an effective therapy in a mice model of AML [40]; however data from clinical studies has not yet been presented.

Taken together, our results with MM cell lines and fresh MM biopsy cells are in agreement with several recent genome-wide studies implicating Polycomb-mediated gene silencing in the development of other types of malignancy [10], [11]. During embryogenesis the PcG proteins constitute an important part of the epigenetic memory by repressing lineage-specific developmental genes. During normal differentiation PcG proteins become redistributed whereby lineage-specific genes are expressed and pluripotency genes become silenced [41], [42], [43]. The fact that an epigenetic mark maintaining self-renewal in embryonic stem cells is found in cancer supports the hypothesis that cancer cells share features with normal stem cells. This observation has previously been limited to tumors possessing a poorly differentiated phenotype [10], [44]. Using integrative genomics and experimental data, we here emphasize that Polycomb gene silencing is associated with what is considered a differentiated plasmablast/plasma cell tumor. In general, terminal differentiation is tightly linked to an irreversible arrest in G1/G0 phase of the cell cycle [45]. Our finding of the underexpressed gene signature in MM suggests that Polycomb silencing enables expansion of differentiated B cells, which retain their ability to divide in conjunction with a differentiated phenotype. In acute leukemia cells, in which an early block in differentiation leads to malignancy, LBH589 induces differentiation [25]. It is therefore of interest to note that the reduction of growth in MM by LBH589 is the result of cell cycle arrest [39], fulfilling at least one of the criteria of differentiation. Additional studies are required to dissect the functional role of the repressed gene signature in maintaining proliferation in MM, and the biological consequences of its reactivation.

The existence of the silenced gene expression profile in MGUS, recently proposed to precede MM in majority of the cases [46], [47], [48], suggests that aberrant Polycomb-mediated gene silencing might be an early event during MM development. Although a small subpopulation of clonogenic CD138 negative B cells are suggested to function as cancer stem cells in MM [49], they are unlikely to have an impact on the underexpressed gene profile, when analyzing the tumor bulk. Instead, the identification of the Polycomb-silenced gene profile in CD138 positive patient samples and cell lines implies that this is a general feature shared by a large proportion of the MM cells. The question, however, remains if MM cells have acquired the Polycomb gene repression signature *de novo* in the late stages of plasma cell differentiation, or if it is a remnant from earlier stages of normal B-cell development, which remains preserved in the MM cells. Though, currently we cannot rule out any of these possibilities, our data argues that maintaining a stemness feature in the plasma cell tumor is important in order to sustain survival and self-renewal of MM.

## Materials and Methods

### Ethics Statements

The study involving human biopsy samples was conducted in accordance with the Declaration of Helsinki and approved by the local ethics committees of Stockholm and Uppsala (Dnr 2004:M-332). Patients gave written informed consent for the sample collection.

In the animal studies animals had free access to food and water and they were housed and treated following the conditions approved by the Ethical Committee for Animal Experiments, VUB (license nr LA1230281).

### Bioinformatic mining

Publically available microarray data were explored using the Oncomine database [21]. Briefly, microarray data were log-transformed, median centered per array, and standard deviation normalized to one per array. Genes underexpressed in MM datasets were compared to literature defined concepts in Oncomine, using the built in t-statistics.

Gene-set enrichment analysis was performed using the GSEA v2.05 software from Broad Institute as previously described [50]. The five selected target genes (CIITA, CXCL12, GATA2, CDH6 and ICSBP/IRF8) were compared with the Mayo clinic data set from Gene Expression Omnibus (GSE6477). The Mayo clinic dataset contains 15 normal bone marrows that were compared to 147 different grades of MM patients.

### Purification of MM cells from patient material and MM cell lines

Heparinised bone marrow samples were obtained from 4 newly diagnosed MM patients. Mononuclear cells were separated by Ficoll-Paque Plus density sedimentation (Amersham Biosciences, Little Chalfont, UK) and were subjected to CD138 immunomagnetic purification according to the manufacturer's protocol (Miltenyi Biotech, Paris, France). The enriched fraction gave a purity of greater than 95%, determined by May-Grünwald-Giemsa staining.

The MM cell lines RPMI 8226 [51] and U-266-1984 [52] (source ATCC and Kenneth Nilsson, Uppsala University, respectively) were maintained in RPMI-1640 AQmedia™ (Sigma®) supplemented with 10% fetal bovine serum (FBS, Sigma®) and antibiotics (penicillin 100 U/ml and streptomycin 50 µg/ml; Sigma®) at 37°C in a humidified 5% CO_2_ in-air atmosphere. Exponentially growing cells were seeded at 2×10^5^ cells/ml (RPMI 8226) or 4×10^5^ (U-266-1984) and incubated overnight before addition of reagents.

### Reagents

LBH589 (Panobinostat, Novartis Pharmaceuticals Inc.) was dissolved in dimethyl sulfoxide (DMSO) and DZNep (National Cancer Institute, Bethesda, MD, USA) was dissolved in dH_2_O. Both chemical inhibitors were used at the indicated concentrations. The concentration of solvent (DMSO or dH_2_O) in each experiment (<0.1%) did not alter the growth and survival of the MM cells (data not shown).

### Chromatin immunoprecipitation (ChIP)

Proteins were cross-linked to DNA using 1% formaldehyde in serum-free medium for 10 min in room temperature. Cross-linking was inhibited by 125 mM glycine. Cells were washed in ice-cold phosphate-buffered saline (PBS), treated with cell lysis buffer (10 mM Tris-HCl pH 8.0, 10 mM NaCl, 0.2% NP-40 and protease inhibitors) for 10 min on ice and cell nuclei were collected at 2500 rpm for 5 min. Nuclei were lysed in RIPA buffer (MOPS (free acid), 0.5 M EDTA pH 8.0, 5N NaCl (to adjust pH to 7.0–7.2), 1% SDS; 10% NP-40, 10% DOC and protease inhibitors) for 10 min on ice and sonicated 3×15 min (30 sec ON/30 sec OFF) at ultrasonic wave output power 320 W in Bioruptor® (Diagenode, Liège, Belgium). Chromatin was collected at 14 000 rpm for 15 min and divided in several fractions. Antibodies used were: anti-H3K9ac (Millipore, 06-942), anti-H3K27me3 (Millipore, 07-449) and IgG (Negative control, OneDay ChIP Kit™Diagenode). Chromatin immunoprecipitation was performed using OneDay ChIP Kit™ (Diagenode, Liège, Belgium) according to the manufacturer's protocol. Precipitated DNA was analyzed by Real Time-qPCR using Platinum® SYBR® Green qPCR SuperMix UDG with Rox (Invitrogen, Carlsbad, CA) and 0.3 mM of each forward and reverse primers. Primer sequences are given in supplementary data (Table S2). The PCR conditions were: 95°C for 2 min followed by 40 cycles of 95°C for 0:30 min and 60°C for 1 min. The run and analysis were performed using Mx3005P instrument and software (Stratagene).

### RNA extraction, cDNA synthesis and Quantitative Real-Time RT-PCR

Total RNA was extracted using TRIzol® (Invitrogen, Carlsbad, CA) according to the manufacturer's protocol. Reverse transcription using random primers (Invitrogen) was performed on 1–2 µg of total RNA using SuperScript™ III Reverse Transcriptase (Invitrogen) according to the manufacturer's protocol.

Each independent quantitative real-time reverse transcriptase PCR (qRT-PCR) reaction contained 5 ng of cDNA, TaqMan® Gene Expression Master Mix (Applied Biosystems, Foster City, CA) and TaqMan® Gene Expression Assays (Applied Biosystems) according to manufacturer's recommendations. The run and analysis were performed using Mx3005P instrument and software (Stratagene).

### Western Blot

At the indicated time points, RPMI 8226 and U-266-1984 cells were harvested, washed with ice-cold PBS and resuspended in lysis buffer containing 1% NP40, 0.1 M Tris-HCl, 0.15 M NaCl, 5 mM EDTA and protease inhibitors (1 mM ZnCl2, 50 mM Na2MoO4, 10 mM NaF, 0.1 mM NaVO3, 1 mM PMSF, 1 mM DTT, 1× complete EDTA-free protease inhibitor (Roche, Mannheim, Germany). Cell lysates were collected at 13 000× g for 10 minutes at 4°C. Proteins were fractionated on NuPAGE® Novex® Bis-Tris gels (Invitrogen, Carlsbad, CA) and transferred onto a nitrocellulose membrane using the iBlot® system (Invitrogen). The membrane was blocked in 5% non-fat dry milk in TBS (10 mM Tris-HCl, pH 7.7, 150 mM NaCl) with 0.1% Tween 20 (TTBS) at room temperature for 1 hour, incubated with the indicated primary antibodies overnight at 4°C, washed in TTBS and incubated for 1 hour at room temperature with the corresponding secondary HRP-conjugated antibodies (Amersham Biosciences) in 5% non-fat dry milk in TTBS. Proteins were visualized using ECL Plus™ Chemiluminescent Detection System. Primary antibodies used were: anti-EZH2 (AC22; #3147, Cell Signaling Technology) and anti-Actin (I-19; sc-1616, Santa Cruz Biotechnology).

### Resazurin assay

RPMI 8226 and U-266-1984 cells were incubated in round bottomed (RPMI 8226) or flat bottomed (U-266-1984) 96-well plates with different concentrations of LBH589 or DZNep. At the indicated time points, 10% AlamarBlue was added to the wells, followed by incubation for 1–3 hours at 37°C in a humidified 5% CO2 in-air atmosphere. Wallac VICTOR Multilabel Counter (Wallac, Turku, Finland) was used for the fluorescence analysis. Resazurin was excited at 530 nm and emitted light was measured at 590 nm. Mean was calculated from triplicate wells and subtracted from mean of blank wells resulting in ΔFluorescence. The relative number of viable cells was expressed as percentage of untreated cells and calculated as 100× ΔFluorescence (treated cells)/ΔFluorescence (untreated cells).

### Apoptosis assay

RPMI 8226 and U-266-1984 were cultured in 6-well plates for 48 and 72 hours in the presence of LBH589 or DZNep. Apoptosis was quantified by Annexin V (AV)-fluorescein isothocyanate (FITC) and PI staining using TACS Annexin V-FITC Apoptosis Kit (R&D Systems, Gaithersburg, MD, USA). Samples were treated according to manufacturer's recommendations and analyzed by flow cytometry (FACScan), presenting apoptotic cells as Annexin V-positive/PI-negative cells and necrotic cells as Annexin V-positive/PI-positive cells.

### Animals

C57BL/KaLwRij mice were purchased from Harlan CPB (Horst, The Netherlands). Mice were used at 6 to 10 weeks of age.

### 5T33MMvv cells

At day 0 C57BL/KalwRij mice were injected with 0.5×10^6^ 5T33MM cells. After 14 days mice were either assigned to a treatment group receiving 10 mg/kg LBH589 (*n* = 4; daily i.p. injection) or to a vehicle group receiving 0.9% NaCl solution (*n* = 4; daily i.p. injection). After 5 days of treatment mice were sacrificed and the BM harvested after flushing out of the femurs and tibiae and crushing out of the vertebrae. The BM cells were suspended in serum-free medium (RPMI 1640; Cambrex, Europe), supplemented with penicillin-streptomycin, glutamine, and minimal essential medium (MEM) nonessential amino acid (NEAA)–pyruvate (Gibco, Life Technologies) and purified by Lympholyte M (Cedarlane, Hornby, ON, Canada) gradient centrifugation at 1000 *g* for 20 minutes, generating enriched 5T33MM cells.

For further enrichment, a negative selection for CD11b positive cells was performed using a MidiMACS magnetic cell separator, LD separation columns and CD11b magnetic microbeads (all from Miltenyi Biotec, Bergisch Gladbach, Germany). 5T33MMvv cells at a concentration of 10^7^ cells per 90 µl MACS buffer (phosphate buffered saline containing 0.5% bovine serum albumin and 2 nM EDTA, pH 7.2) and 10 µl CD11b Microbeads were incubated at 4°C for 15 min. The 5T33MMvv-microbeads mixture was then loaded onto a separation column placed on the magnetic cell separator. After the flow through, the column was washed three times using 500 ml of MACS buffer and the eluted cell fraction was collected for further experiments.

### Assessment of the tumor burden *in vivo*

The 5T33MM cells were injected in C57BL/KalwRij mice as described above. At day 0 mice were either assigned to a treatment group receiving 10 mg/kg LBH589 (*n* = 10; daily i.p. injection), to a vehicle group receiving 0.9% NaCl solution (*n* = 10; daily i.p. injection) or to an untreated, disease-free group (n = 10) as a control group. The animals were sacrificed when they showed signs of morbidity (paralysis). Tumor load was analyzed by means of serum paraprotein concentration and BM plasmocytosis: the first was quantified by electrophoresis and assessment of total protein, the latter by quantification of May Grünwald-Giemsa staining of BM cytospin samples.

### Survival analysis

Survival was studied using Kaplan-Meier analysis. 5T33MM cells were injected as above and at day 0 mice received 10 mg/kg LBH589 (*n* = 12; daily i.p. injection) or vehicle, 0.9% NaCl solution (*n* = 12; daily i.p. injection). An untreated disease-free group (n = 12) was used as a control. Each mouse was sacrificed when it showed signs of morbidity (paralysis).

## Supporting Information

Figure S1 The PRC2 components EED, EZH2 and SUZ12 are overexpressed in MM. (A): MM patients (n = 74) compared to plasma cells (n = 37) and tonsillar tissue (n = 37) (Zhan, Hardin et al. 2002). (B): Smoldering Myeloma patients (n = 12) compared to MGUS patients (n = 44) and normal bone marrow (n = 22) (Zhan, Barlogie et al. 2007). (C): MM stage III patients (n = 34) compared to MM stage I (n = 30) and stage II (n = 38) (Agnelli, Fabris et al. 2007). Analysis was performed on normalized expression units, using the built in t-statistics (Rhodes, Yu et al. 2004).
(0.99 MB TIF)

Figure S2 The PRC2 targets CIITA, GATA2, CDH6, CXCL12 and ICSBP/IRF8 are underexpressed in MM. Target genes were defined by Bracken et al. (Bracken, Dietrich et al. 2006) in embryonic fibroblasts and their expression pattern examined using Oncomine. (A): MM patients (n = 74) compared to plasma cells (n = 37) and tonsillar tissue (n = 37) (Zhan, Hardin et al. 2002). (B): Smoldering Myeloma patients (n = 12) compared to MGUS patients (n = 44) and normal bone marrow (n = 22) (Zhan, Barlogie et al. 2007). (C): MM stage III patients (n = 34) compared to MM stage I (n = 30) and stage II (n = 38) (Agnelli, Fabris et al. 2007). Analysis was performed on normalized expression units, using the built in t-statistics (Rhodes, Yu et al. 2004).
(1.51 MB TIF)

Figure S3 The H3K27-tri-methylated CIITA, GATA2, CDH6, CXCL12 and ICSBP/IRF8 are enriched among genes underexpressed in MM. Enrichment profile generated after a gene-set enrichment (GSE) analysis of the five PRC2 target genes (CIITA, GATA2, CDH6, CXCL12 and ICSBP/IRF8) when compared to Mayo clinic dataset (Chng, Kumar et al. 2007) (GSE6477) using software GSEA v 2.05 with FDR q-value 0.0181.
(0.68 MB TIF)

Figure S4 DZNep and LBH589 deplete EZH2 protein expression in a concentration-dependent manner. (A): The RPMI 8226 cells and (B): The U-266-1984 cells were treated for 48 hours with 0.5, 2.5 and 10 µM DZNep; (C): the RPMI 8226 cells were treated for 72 hours with 4, 10 and 20 nM LBH589 and (D): the U-266-1984 cells were treated for 24 hours with 4, 20 and 100 nM LBH589 followed by western blot analysis using anti-EZH2 antibody. Actin was used to control for equal loading.
(0.14 MB TIF)

Figure S5 DZNep and LBH589 reduce growth of the RPMI 8226 and U-266-1982 cells. (A) and (B): RPMI 8226 and U-266-1984 were treated with the indicated concentrations of DZNep (48 hours) and LBH589 (24 hours) followed by AlamarBlue assay. At least 2 experiments were performed in triplicates; data are presented as mean percentage of control ±SD.
(0.08 MB TIF)

Figure S6 DZNep and LBH589 have mild effect on growth of the normal SK 1064 cells and PBMC. (A) and (B): SK 1064 and PBMC were treated with the indicated concentrations of DZNep (48 hours) and LBH589 (48 hours) followed by resazurin assay. Experiment was performed in triplicates; data are presented as mean percentage of control ±SD. Peripheral blood mononuclear cells (PBMC) were isolated by Ficoll-Hypaque separation of buffy coats from healthy donors.
(0.09 MB TIF)

Table S1 H3K27me3 targets among the 10% most under expressed genes in MM, MGUS and MM stage III, corresponding to figure 1a, 1b and 1c. Complete gene list of H3K27me3 targets (Bracken, Dietrich et al. 2006) among the 10% most under expressed genes in MM (Zhan, Hardin et al. 2002), MGUS (Zhan, Barlogie et al. 2007) and MM stage III (Agnelli, Fabris et al. 2007), corresponding to figure 1a, 1b and 1c.
(0.33 MB XLS)

Table S2 Primers used in real-time qPCR for chromatin immunoprecipitation (ChIP). Primers used in real-time qPCR for chromatin immunoprecipitation (ChIP).
(0.03 MB DOC)
