# Supplementary figures and images for: Polycomb Target Genes Are Silenced in Multiple Myeloma
Source: PLoS One. 2010 Jul 9;5(7):e11483. doi: 10.1371/journal.pone.0011483 (PMC2901331; doi:10.1371/journal.pone.0011483)

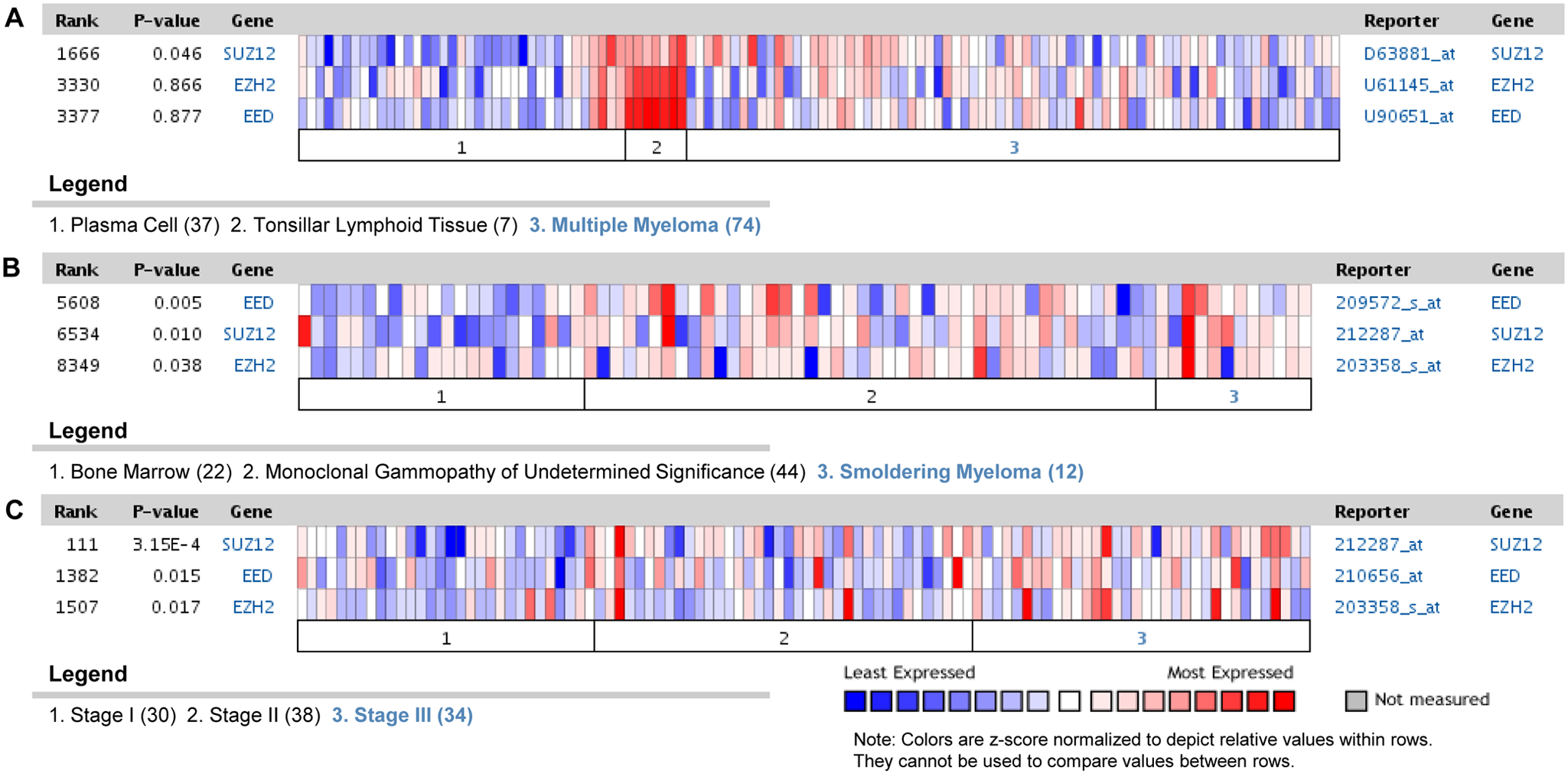

Supplement: Figure S1 — The PRC2 components EED, EZH2 and SUZ12 are overexpressed in MM. (A): MM patients (n = 74) compared to plasma cells (n = 37) and tonsillar tissue (n = 37) (Zhan, Hardin et al. 2002). (B): Smoldering Myeloma patients (n = 12) compared to MGUS patients (n = 44) and normal bone marrow (n = 22) (Zhan, Barlogie et al. 2007). (C): MM stage III patients (n = 34) compared to MM stage I (n = 30) and stage II (n = 38) (Agnelli, Fabris et al. 2007). Analysis was performed on normalized expression units, using the built in t-statistics (Rhodes, Yu et al. 2004). (0.99 MB TIF) [file pone.0011483.s001.tif]

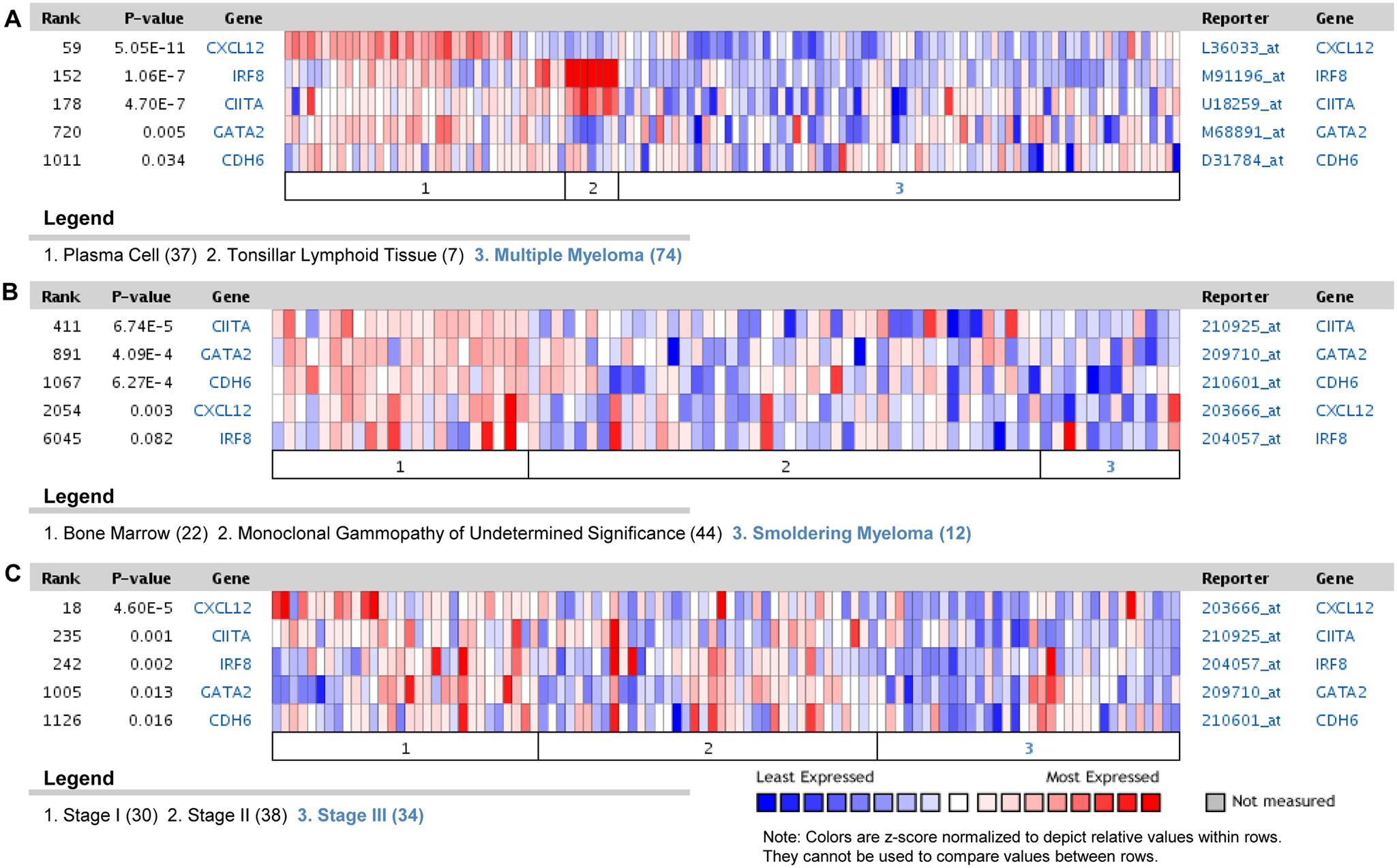

Supplement: Figure S2 — The PRC2 targets CIITA, GATA2, CDH6, CXCL12 and ICSBP/IRF8 are underexpressed in MM. Target genes were defined by Bracken et al. (Bracken, Dietrich et al. 2006) in embryonic fibroblasts and their expression pattern examined using Oncomine. (A): MM patients (n = 74) compared to plasma cells (n = 37) and tonsillar tissue (n = 37) (Zhan, Hardin et al. 2002). (B): Smoldering Myeloma patients (n = 12) compared to MGUS patients (n = 44) and normal bone marrow (n = 22) (Zhan, Barlogie et al. 2007). (C): MM stage III patients (n = 34) compared to MM stage I (n = 30) and stage II (n = 38) (Agnelli, Fabris et al. 2007). Analysis was performed on normalized expression units, using the built in t-statistics (Rhodes, Yu et al. 2004). (1.51 MB TIF) [file pone.0011483.s002.tif]

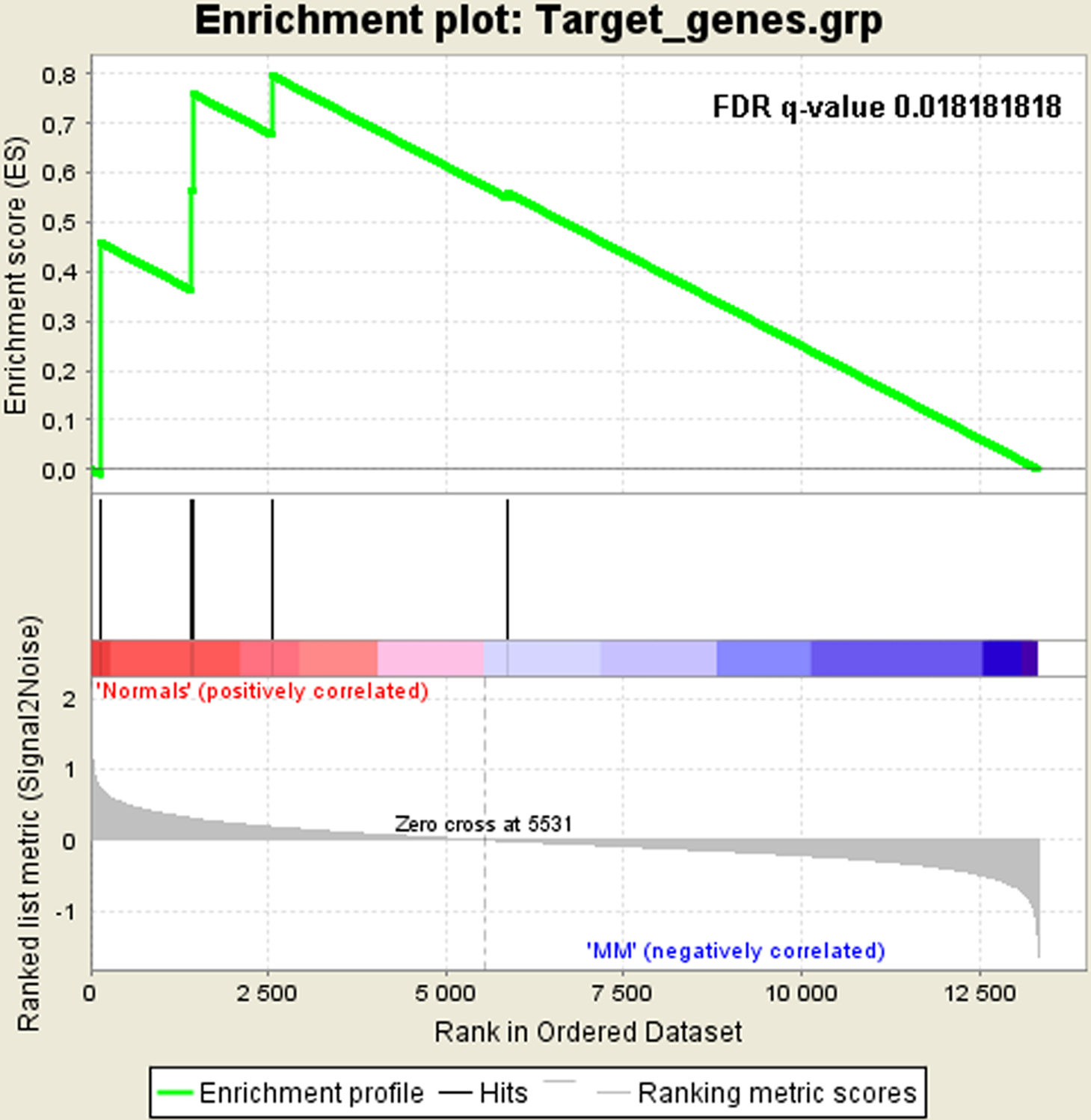

Supplement: Figure S3 — The H3K27-tri-methylated CIITA, GATA2, CDH6, CXCL12 and ICSBP/IRF8 are enriched among genes underexpressed in MM. Enrichment profile generated after a gene-set enrichment (GSE) analysis of the five PRC2 target genes (CIITA, GATA2, CDH6, CXCL12 and ICSBP/IRF8) when compared to Mayo clinic dataset (Chng, Kumar et al. 2007) (GSE6477) using software GSEA v 2.05 with FDR q-value 0.0181. (0.68 MB TIF) [file pone.0011483.s003.tif]

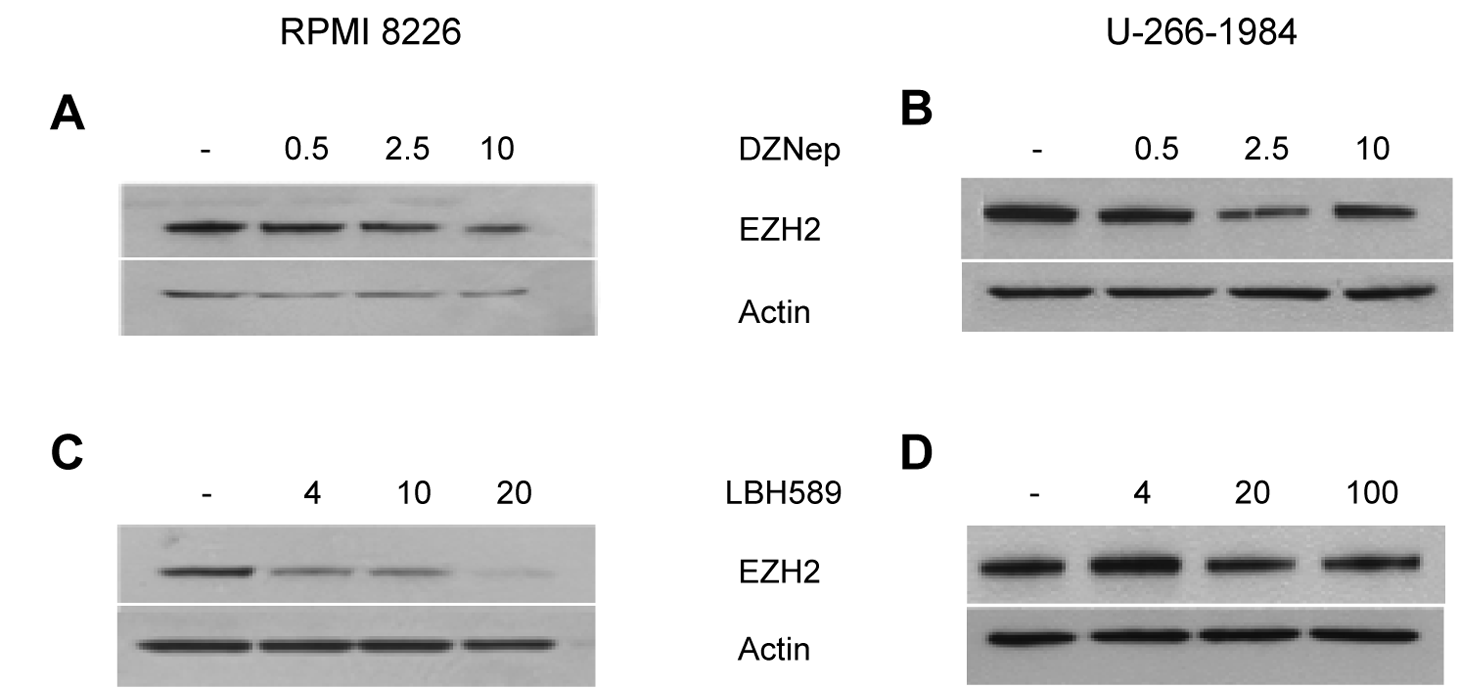

Supplement: Figure S4 — DZNep and LBH589 deplete EZH2 protein expression in a concentration-dependent manner. (A): The RPMI 8226 cells and (B): The U-266-1984 cells were treated for 48 hours with 0.5, 2.5 and 10 µM DZNep; (C): the RPMI 8226 cells were treated for 72 hours with 4, 10 and 20 nM LBH589 and (D): the U-266-1984 cells were treated for 24 hours with 4, 20 and 100 nM LBH589 followed by western blot analysis using anti-EZH2 antibody. Actin was used to control for equal loading. (0.14 MB TIF) [file pone.0011483.s004.tif]

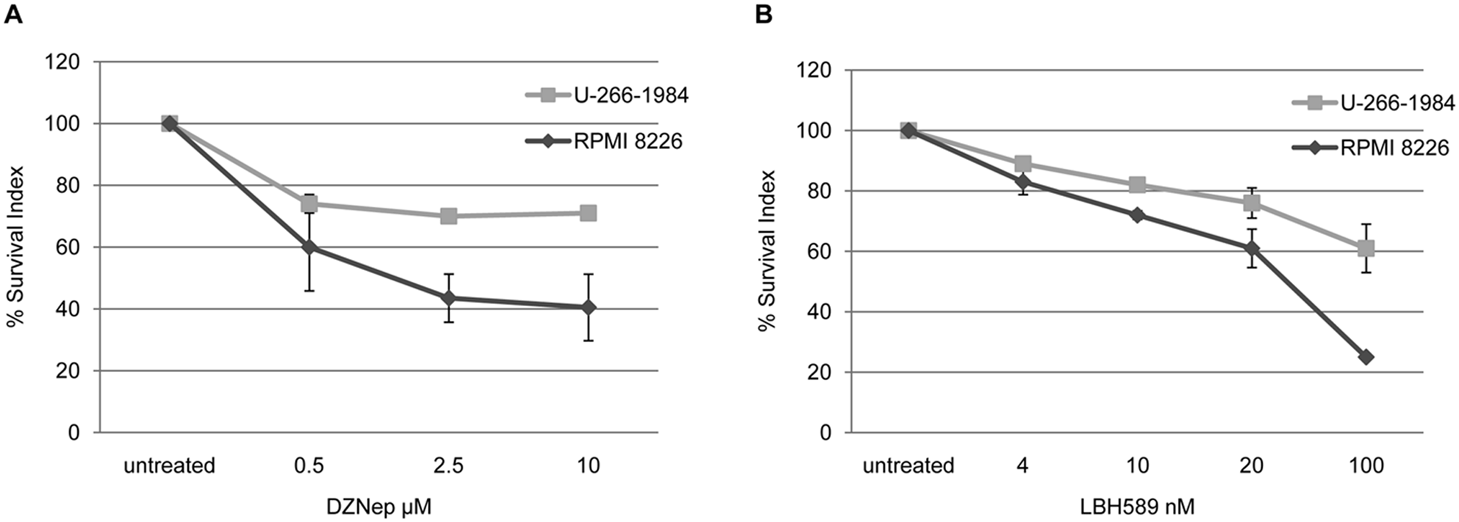

Supplement: Figure S5 — DZNep and LBH589 reduce growth of the RPMI 8226 and U-266-1982 cells. (A) and (B): RPMI 8226 and U-266-1984 were treated with the indicated concentrations of DZNep (48 hours) and LBH589 (24 hours) followed by AlamarBlue assay. At least 2 experiments were performed in triplicates; data are presented as mean percentage of control ±SD. (0.08 MB TIF) [file pone.0011483.s005.tif]

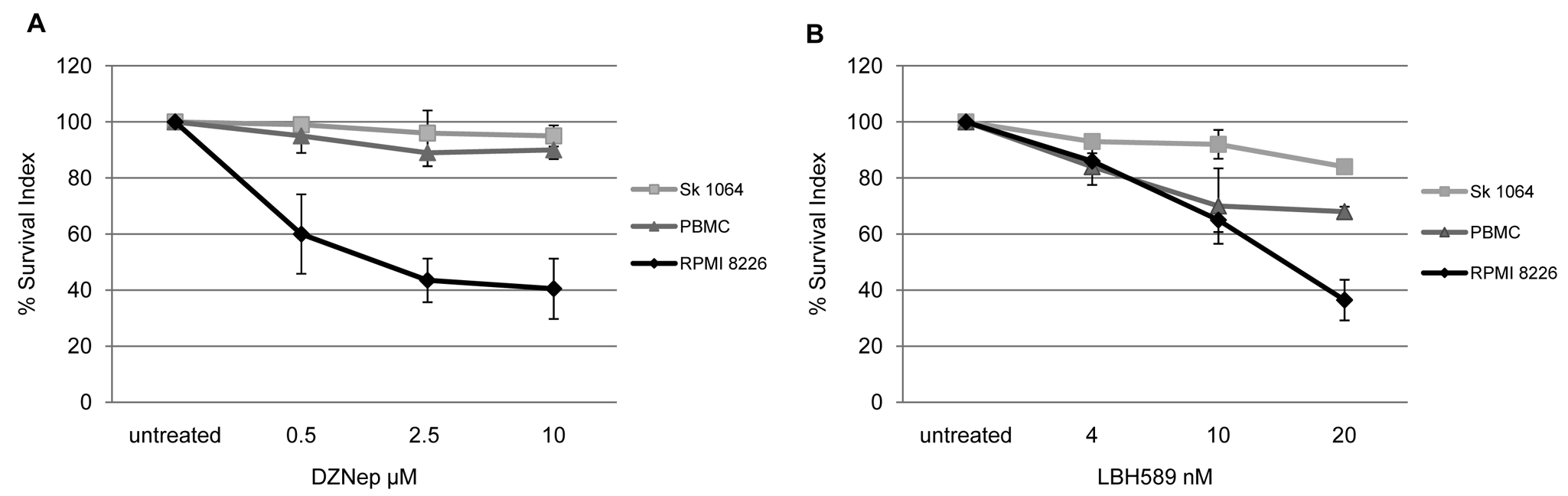

Supplement: Figure S6 — DZNep and LBH589 have mild effect on growth of the normal SK 1064 cells and PBMC. (A) and (B): SK 1064 and PBMC were treated with the indicated concentrations of DZNep (48 hours) and LBH589 (48 hours) followed by resazurin assay. Experiment was performed in triplicates; data are presented as mean percentage of control ±SD. Peripheral blood mononuclear cells (PBMC) were isolated by Ficoll-Hypaque separation of buffy coats from healthy donors. (0.09 MB TIF) [file pone.0011483.s006.tif]
