# Supplementary material for: Polycomb Target Genes Are Silenced in Multiple Myeloma
Source: PLoS One. 2010 Jul 9;5(7):e11483. doi: 10.1371/journal.pone.0011483 (PMC2901331; doi:10.1371/journal.pone.0011483)
Supplement: Table S2 — Primers used in real-time qPCR for chromatin immunoprecipitation (ChIP). Primers used in real-time qPCR for chromatin immunoprecipitation (ChIP). (0.03 MB DOC) [file pone.0011483.s008.doc]

**Table S2. Primers used in real-time qPCR for chromatin immunoprecipitation (ChIP).**

| **Gene** | **Forward** | **Reverse** |
| --- | --- | --- |
| SERPINA1 (ENSG00000197249) | GGCTCAAGCTGGCATTCCT | GGCTTA ATCACGCACTGAGCTTA |
| RPL30 (ENSG00000156482) | CAAGGCAAAGCGAAATTGGT | GCCCGTTCAGTCTCTTCGATT |
| GAPDH^(1)^ (ENSG00000111640) | TACTAGCGGTTTTACGGGCG | TCGAACAGGAGGAGCAGAGAGCGA |
| CIITA (ENSG00000179583) | GAGGTGGCAAAAGCCTAGAA | GGGAGCAGAAACGGAAGACT |
| CXCL12 (ENSG00000107562) | GTATCCGGAGGGCTAAGCA | CGACCCAAAGAGCTGAGAAC |
| GATA2 (ENSG00000179348) | ATCAGTGAGTGCGTGTGCTC | GCTCAGAAAGCTTGGGACAC |
| CDH6 (ENSG00000113361) | GCACTGGGCCTCTTTAACAA | TTGTGGGCACACACTGATTT |
| ICSBP/IRF8(ENSG00000140968) | GCGGCAGGTAGGCACAGT | CGGGGACATTACGGTAGGTA |
| INK4A ^(2)^ (ENSG00000147889) | CTGGAGGACGAAGTTTGC | AGGAGGAGGTCTGTGATTAC |

1. Varambally, S., Q. Cao, et al. (2008). "Genomic loss of microRNA-101 leads to overexpression of histone methyltransferase EZH2 in cancer." Science 322(5908): 1695-9.
2. Kia, S. K., M. M. Gorski, et al. (2008). "SWI/SNF mediates polycomb eviction and epigenetic reprogramming of the INK4b-ARF-INK4a locus." Mol Cell Biol 28(10): 3457-64.
